# Supplementary material for: Extracellular DNA traps in a ctenophore demonstrate immune cell behaviors in a non-bilaterian
Source: Nat Commun. 2024 Apr 6;15:2990. doi: 10.1038/s41467-024-46807-6 (PMC10998917; doi:10.1038/s41467-024-46807-6)
Supplement: Supplementary file 2 — Description of Additional Supplementary Files [file 41467_2024_46807_MOESM2_ESM.pdf]

Description of Additional Supplementary Files

Supplementary Movie 1: Video of DIC timelapse images of isolated *Mnemiopsis* cells *in vitro*. An amoebocyte-like granular cell (arrowhead) and highly motile stellate cell (arrow) are visible.

Supplementary Movie 2: Video of DIC timelapse images of an isolated *Mnemiopsis* stellate cell *in vitro*. The cell initially has two large processes, then absorbs them and subsequently produces several additional processes. The cell then begins to crawl out of view.

Supplementary Movie 3: Merged brightfield and fluorescent video of a live *Mnemiopsis* motile, stellate cell that is phagocytosing fluorescent *E. coli* (red). Scale bar is 10 µm.

Supplementary Movie 4: Merged brightfield and fluorescent video of a live *Mnemiopsis* motile, stellate cell that is phagocytosing fluorescent *E. coli* (green). *Mnemiopsis* cells were labeled with Lysotracker Red. Scale bar is 10 µm.

Supplementary Movie 5: Merged brightfield and fluorescent video of a live *Mnemiopsis* motile, stellate cell undergoing ETosis after *in vitro* exposure to pHrodo-*E. coli*. The cell moves into view, retracts its processes, spins, and exudes its nuclear material. DNA (Hoechst, blue), pHrodo-*E. coli* (red)

Supplementary Movie 6: 3D reconstruction of confocal stack of *Mnemiopsis* extracellular DNA traps. DNA (Hoechst, white) is extracellular, in filamentous “nets”; lysosomal marker (Lysotracker, green) denotes cellular debris; bacteria are ensnared in the “nets” (pHrodo-*E. coli*, red)
