# Supplementary material for: Extracellular DNA traps in a ctenophore demonstrate immune cell behaviors in a non-bilaterian
Source: Nat Commun. 2024 Apr 6;15:2990. doi: 10.1038/s41467-024-46807-6 (PMC10998917; doi:10.1038/s41467-024-46807-6)
Supplement: Supplementary file 9 — Reporting Summary [file 41467_2024_46807_MOESM9_ESM.pdf]

nature portfolio

Corresponding author(s): Lauren E Vandepas

Last updated by author(s): Feb 27, 2024

Reporting Summary

Nature Portfolio wishes to improve the reproducibility of the work that we publish. This form provides structure for consistency and transparency in reporting. For further information on Nature Portfolio policies, see our Editorial Policies and the Editorial Policy Checklist.

Statistics

For all statistical analyses, confirm that the following items are present in the figure legend, table legend, main text, or Methods section.

n/a	Confirmed	
☐	☒	The exact sample size (*n*) for each experimental group/condition, given as a discrete number and unit of measurement
☐	☒	A statement on whether measurements were taken from distinct samples or whether the same sample was measured repeatedly
☐	☒	The statistical test(s) used AND whether they are one- or two-sided<br>*Only common tests should be described solely by name; describe more complex techniques in the Methods section.*
☒	☐	A description of all covariates tested
☒	☐	A description of any assumptions or corrections, such as tests of normality and adjustment for multiple comparisons
☐	☒	A full description of the statistical parameters including central tendency (e.g. means) or other basic estimates (e.g. regression coefficient) AND variation (e.g. standard deviation) or associated estimates of uncertainty (e.g. confidence intervals)
☐	☒	For null hypothesis testing, the test statistic (e.g. *F*, *t*, *r*) with confidence intervals, effect sizes, degrees of freedom and *P* value noted<br>*Give P values as exact values whenever suitable.*
☒	☐	For Bayesian analysis, information on the choice of priors and Markov chain Monte Carlo settings
☒	☐	For hierarchical and complex designs, identification of the appropriate level for tests and full reporting of outcomes
☒	☐	Estimates of effect sizes (e.g. Cohen's *d*, Pearson's *r*), indicating how they were calculated

Our web collection on statistics for biologists contains articles on many of the points above.

Software and code

Policy information about availability of computer code

Data collection	Imaging: automated imaging plate reader Cytation 3 (Biotek, software Gen5 v4.2); Nikon Ti (Eclipse) inverted microscope with Ultraview Spinning Disc (CSU-X1) confocal scanner (Perkin Elmer). Images were captured with an Orca-ER Camera using Volocity (Quorum technologies);
Data analysis	CellProfiler (v4.1.3); R (v4.0.5) software with tidyverse (v1.3.1), dplyr (v1.0.7) and readr (v1.4.0) packages; GraphPad Prism (v9.2.0); https://github.com/carolinestefani/ETosis-and-death-automated-pipeline

For manuscripts utilizing custom algorithms or software that are central to the research but not yet described in published literature, software must be made available to editors and reviewers. We strongly encourage code deposition in a community repository (e.g. GitHub). See the Nature Portfolio guidelines for submitting code & software for further information.

Data

Policy information about availability of data

All manuscripts must include a data availability statement. This statement should provide the following information, where applicable:

- Accession codes, unique identifiers, or web links for publicly available datasets
- A description of any restrictions on data availability
- For clinical datasets or third party data, please ensure that the statement adheres to our policy

All data supporting the findings of this study are available within the paper and its paired Supplementary Information. The novel imaging analysis pipeline for quantification of cell death and ETosis can be found at https://github.com/carolinestefani/ETosis-and-death-automated-pipeline

Research involving human participants, their data, or biological material

Policy information about studies with human participants or human data. See also policy information about sex, gender (identity/presentation), and sexual orientation and race, ethnicity and racism.

Reporting on sex and gender	N/A
Reporting on race, ethnicity, or other socially relevant groupings	N/A
Population characteristics	N/A
Recruitment	N/A
Ethics oversight	N/A

Note that full information on the approval of the study protocol must also be provided in the manuscript.

Field-specific reporting

Please select the one below that is the best fit for your research. If you are not sure, read the appropriate sections before making your selection.

☒ Life sciences ☐ Behavioural & social sciences ☐ Ecological, evolutionary & environmental sciences

For a reference copy of the document with all sections, see nature.com/documents/nr-reporting-summary-flat.pdf

Life sciences study design

All studies must disclose on these points even when the disclosure is negative.

Sample size	For stimulation and quantification of ETosis, hundreds of thousands of cells were isolated from individual animals and plated in 96 well plates. Number of animals and replicates experiment are indicated in the figure legend for each figure panel.
Data exclusions	Data from animals showing high death in untreated conditions were excluded
Replication	Number of animals and replicates experiment are indicated in the figure legend for each figure panel.
Randomization	Animal selection was randomized. Individual ctenophores and oysters were selected from group tanks. No mutant strains were used.
Blinding	Blinding was used for counting the number of ETotic cells in Figure 2E.

Reporting for specific materials, systems and methods

We require information from authors about some types of materials, experimental systems and methods used in many studies. Here, indicate whether each material, system or method listed is relevant to your study. If you are not sure if a list item applies to your research, read the appropriate section before selecting a response.

Materials & experimental systems

n/a	Involved in the study	
☐	☒	Antibodies
☒	☐	Eukaryotic cell lines
☒	☐	Palaeontology and archaeology
☐	☒	Animals and other organisms
☒	☐	Clinical data
☒	☐	Dual use research of concern
☒	☐	Plants

Methods

n/a	Involved in the study	
☒	☐	ChIP-seq
☒	☐	Flow cytometry
☒	☐	MRI-based neuroimaging

Antibodies

Antibodies used	histone labeling was performed with mouse anti-histone H11-4 (EMD Millipore, cat. MAB3422) at a dilution of 1:200 and goat anti-mouse Alexa Fluor 488 (Thermo Fisher Scientific, cat. A28175) at a dilution of 1:500.

Validation	We applied negative staining controls to validate both primary and secondary antibody labeling specificities for Mnemiopsis leidyi nuclei. This validation included examination of samples by fluorescence microscopy where primary antibody alone was applied to cells, (without a secondary antibody) or where secondary antibody was applied in the absence of primary antibody. No nonspecific labeling was detected for either the primary or secondary antibody negative controls.<br>The vendor web site states: "The antibody binds specifically to an antigen determinant, which is present on all five histone proteins. The antibody recognizes the histones H1, H2A, H2B, H3 and H4 with comparable sensitivity." References that have utilized this pan-histone antibody include assays examining ET production: doi: 10.1371/journal.pone.0075141

Animals and other research organisms

Policy information about studies involving animals; ARRIVE guidelines recommended for reporting animal research, and Sex and Gender in Research

Laboratory animals	Adult Mnemiopsis leidyi (generation F32) were used and maintained as described previously: DOI: 10.1038/s41596-022-00702-w
Wild animals	Adult giant Pacific oyster (Crassostrea gigas) were obtained from Taylor Shellfish Farms. The oysters were removed from common aquaculture tanks at the shellfish farm and given to LEV to be transported in a refrigerated cooler from the aquaculture facility in Shelton, WA, USA to Manchester, WA, USA. The oysters were maintained in flowing seawater at ambient temperature in communal tanks. One day prior to ETosis assays, oysters were transported from the marine laboratories in Manchester, WA, USA to the Benaroya Research Institute in Seattle, WA and maintained in synthetic seawater with aeration. Following non-lethal hemolymph collection via a hole drilled into the left shell, the oysters were marked and returned to their original common tanks.<br>While it is not possible to know the exact age of the oysters that were acquired from the aquaculture facility, it is certain that they were older than 3 years of age (adult mature oysters, in contrast to juveniles/spat).
Reporting on sex	Both study organisms are hermaphrodites.
Field-collected samples	No field samples were collected for this study.
Ethics oversight	No ethical approval or guidance was required because all animals used in this study were non-cephalopod invertebrates.

Note that full information on the approval of the study protocol must also be provided in the manuscript.

Plants

Seed stocks	N/A
Novel plant genotypes	N/A
Authentication	N/A
